# Comparative analysis of the complete mitochondrial genomes in two limpets from Lottiidae (Gastropoda: Patellogastropoda): rare irregular gene rearrangement within Gastropoda

**DOI:** 10.1038/s41598-020-76410-w

**Published:** 2020-11-06

**Authors:** Jian-tong Feng, Ya-hong Guo, Cheng-rui Yan, Ying-ying Ye, Ji-ji Li, Bao-ying Guo, Zhen-ming Lü

**Affiliations:** 1 National Engineering Research Center for Marine Aquaculture, Zhejiang Ocean University, Zhoushan, 316022 China; 2 National Engineering Laboratory of Marine Germplasm Resources Exploration and Utilization, Zhejiang Ocean University, Zhoushan, 316022 China

**Keywords:** Evolution, Molecular biology

## Abstract

To improve the systematics and taxonomy of Patellogastropoda within the evolution of gastropods, we determined the complete mitochondrial genome sequences of *Lottia goshimai* and *Nipponacmea fuscoviridis* in the family Lottiidae, which presented sizes of 18,192 bp and 18,720 bp, respectively. In addition to 37 common genes among metazoa, we observed duplication of the *trnM* gene in *L. goshimai* and the *trnM* and *trnW* genes in *N. fuscoviridis*. The highest A + T contents of the two species were found within protein-coding genes (59.95% and 54.55%), followed by rRNAs (56.50% and 52.44%) and tRNAs (56.42% and 52.41%). *trnS1* and *trnS2* could not form the canonical cloverleaf secondary structure due to the lack of a dihydrouracil arm in both species. The gene arrangements in all Patellogastropoda compared with those of ancestral gastropods showed different levels of gene rearrangement, including the shuffling, translocation and inversion of single genes or gene fragments. This kind of irregular rearrangement is particularly obvious in the Lottiidae family. The results of phylogenetic and gene rearrangement analyses showed that *L. goshimai* and *Lottia digitalis* clustered into one group, which in turn clustered with *N. fuscoviridis* in Patellogastropoda. This study demonstrates the significance of complete mitogenomes for phylogenetic analysis and enhances our understanding of the evolution of Patellogastropoda.

## Introduction

The order Patellogastropoda (common name, true limpets) consists of the most primitive gastropod molluscs, which inhabit intertidal rocky shores worldwide, from tropical to polar regions^1,2^. Most species from this group feed by scraping a fine film of microalgae from what appears to be a bare rock surface. Their shells have the appearance of hats of different sizes, generally reaching no more than 20 cm^3,4^. These limpets, which are ecologically important in coastal regions, separated from other gastropods early in molluscan evolution^5^. They can be used in marine ecotoxicology research because of their wide distribution, and their gametes are available throughout the year^6^. This group is characterized by intraspecific polyphenism, the existence of cryptic species, and the intraspecific variation of characteristics such as shell morphology and colour. Therefore, it is difficult to identify species in this group by traditional methods alone^7–10^. The phylogenetic analysis of Patellogastropoda has generally focussed on Caenogastropoda, Neomphalina, Vetigastropoda, Neritimorpha and Heterobranchia. The phylogenetic position of the group and the evolutionary relationships among families of true limpets have been highly controversial^11,12^.

*Nipponacmea fuscoviridis* (Teramachi, 1949) and *Lottia goshimai* (Nakayama, Sasaki & T. Nakano, 2017) both belong to the family Lottiidae. *N. fuscoviridis* commonly appears in temperate areas around the Japanese islands and the southeastern coastal region of China^13^. Species of this genus are common along the Asian coast of the Pacific Ocean from Vietnam to Russia^14^. *L. goshimai* was previously thought to be an intraspecific variant of the northern population of *N. fuscoviridis* and was later proven to be a new species^15^; thus, there have been few studies on this species. Its developmental stages and gene expression were studied by Wang et al.^16–18^.

The complete mitochondrial genome provides more information than individual genes. It exhibits the characteristics of maternal inheritance, a high evolutionary rate and a relatively low intermolecular recombination rate, and it is becoming increasingly common for mitochondrial genomes to be used for phylogenetic reconstruction^19–22^. The circular mitochondrial genome of gastropods generally contains 37 genes (22 transfer RNA genes, two ribosomal RNA genes, 13 protein-coding genes) and a noncoding control region. Nevertheless, Lottiidae species seem to be an exception, exhibiting different numbers of tRNA genes^23^.

In the present study, two mitochondrial genomes (*L. digitalis* and *N. fuscoviridis*) from the Lottiidae family were sequenced, annotated and compared to the other available genomes from Patellogastropoda. We analysed the main characteristics of the newly generated mitogenomes, such as their nucleotide composition, codon usage and the secondary structure of their tRNAs. Complete mitogenome sequences from six subclasses of Gastropoda were downloaded from the GenBank database to reconstruct the phylogenetic tree. The results will help us to obtain further insight into the evolutionary relationships within Patellogastropoda.

## Results and discussion

### Characteristics, structure and organization of the mitogenomes

**Figure 1:**
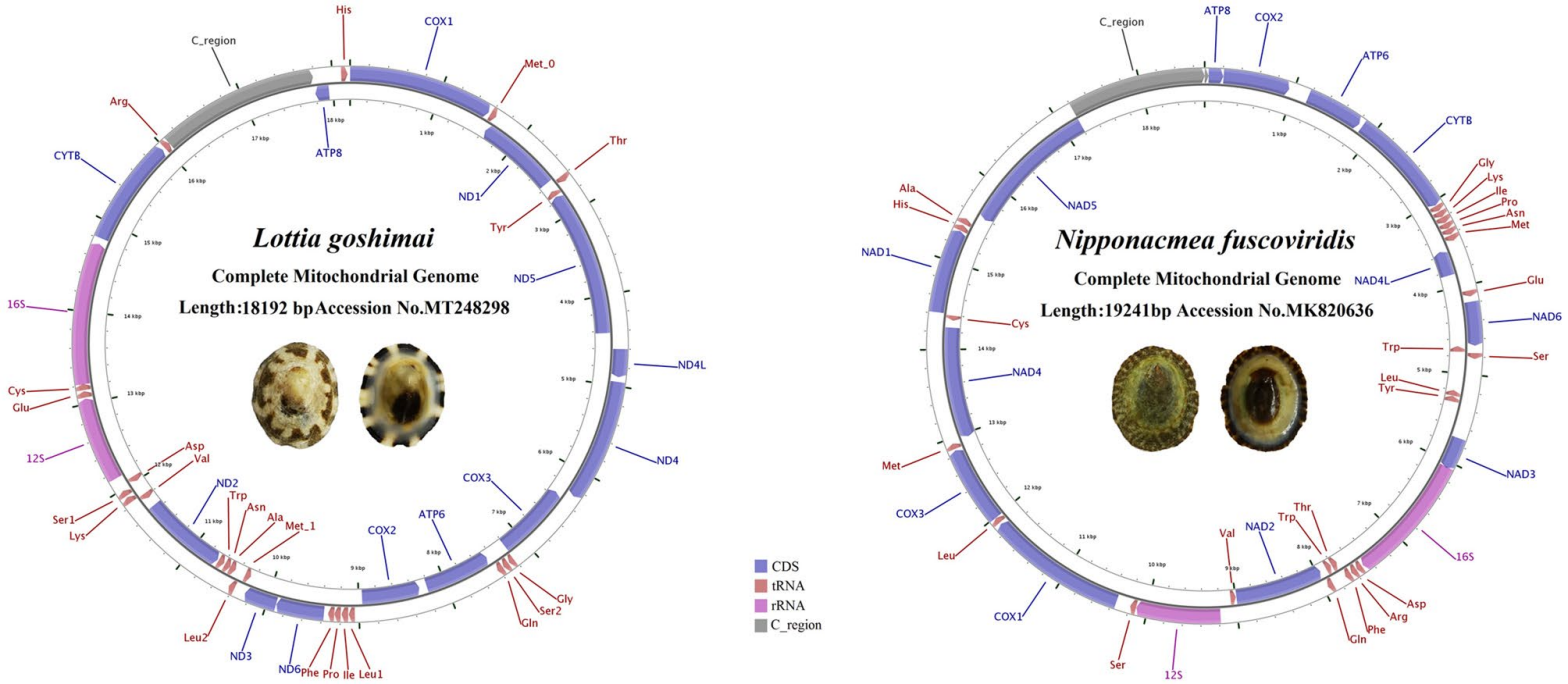
Maps of the mitochondrial genomes of two Lottiidae species. The direction of gene transcription is indicated by the arrows.

The gene arrangements found within Patellogastropoda mitochondrial genomes have been relatively conservative, but those of Lottiidae differ to some extent. The comparison of the two newly sequenced mitogenomes with a reported mitogenome from Lottiidae revealed the rearrangement of gene positions and structures. The complete mitochondrial genome sequences of *L. goshimai* and *N. fuscoviridis* were 18,192 bp and 18,720 bp, respectively (GenBank accessions MT248298 and MK395167) (Fig. 1, Table 1). Both circular mitochondrial genomes of the species contained 13 PCGs, 2 rRNA genes (12S rRNA and 16S rRNA), 22 putative tRNA genes and a control region (CR). Compared to the fragment of the genome previously published, we found an additional *trnM* gene in both species and additional *trnW* gene *in N. fuscoviridis*.

**Table 1:** List of species analysed in this study with their GenBank accession numbers.

Subclass	Family	Species	Size (bp)	Accession no.
Caenogastropoda	Muricidae	*Concholepas concholepas*	15,495	NC_017886
		*Rapana venosa*	15,272	EU170053
	Conidae	*Conus tribblei*	15,570	NC027957
		*Conus tulipa*	15,756	KR006970
	Turridae	*Turricula nelliae spuria*	16,453	MK251986
	Xenophoridae	*Onustus exutus*	16,043	MK327366
	Pomatiopsidae	*Oncomelania hupensis robertsoni*	15,188	LC276228
		*Oncomelania hupensis nosophora*	15,182	LC276226
		*Oncomelania quadrasi*	15,184	LC276227
	Turritellidae	*Turritella bacillum*	15,868	NC_029717
	Epitoniidae	*Epitonium scalare*	15,143	MK251987
Neomphaliones	Bathysciadiidae	*Bathysciadiidae sp.*	17,238	MH837532
	Cocculinidae	*Coccocrater sp.*	13,983	MH837535
		*Cocculina subcompressa*	18,167	MH837536
Vetigastropoda	Turbinidae	*Angaria delphinus*	19,554	NC_031860
		*Angaria neglecta*	19,470	NC_028707
		*Astralium haematragum*	16,310	NC_031858
		*Bolma rugosa*	17,432	NC_029366
		*Lunella aff. Cinereal*	17,670	KF700096
		*Lunella granulate*	17,190	NC_031857
	Tegulidae	*Tegula brunnea*	17,690	NC_016954
		*Tegula lividomaculata*	17,375	NC_029367
		*Tectus pyramis*	18,439	MF138911
	Trochidae	*Gibbula umbilicalis*	16,277	NC_035682
		*Stomatella planulata*	17,151	NC_031861
		*Umbonium thomasi*	15,998	MH729882
	Haliotidae	*Haliotis rufescens*	16,646	NC_036928
		*Haliotis laevigata*	16,545	NC_024562
		*Haliotis rubra*	16,907	AY588938
		*Haliotis tuberculata*	16,521	FJ599667
	Phasianellidae	*Phasianella solida*	16,698	NC_028709
Neritimorpha	Neritidae	*Clithon retropictus*	15,802	NC_037238
		*Nerita albicilla*	15,314	MK516738
		*Nerita yoldii*	15,719	MK395169
		*Nerita fulgurans*	15,343	KF728888
		*Nerita tessellata*	15,741	KF728889
		*Nerita versicolor*	15,866	KF728890
		*Nerita melanotragus*	15,261	GU810158
Heterobranchia	Aplysiidae	*Aplysia californica*	14,117	AY569552
		*Aplysia dactylomela*	14,128	DQ991927
		*Aplysia kurodai*	14,131	KF148053
	Polyceridae	*Roboastra europaea*	14,472	NC_004321
	Siphonariidae	*Siphonaria pectinate*	14,065	AY345049
	Volvatellidae	*Ascobulla fragilis*	14,745	AY345022
	Placobranchidae	*Elysia cornigera*	14,118	NC_035489
		*Elysia timida*	14,088	NC_035490
	Onchidiidae	*Onchidella celtica*	14,150	AY345048
		*Platevindex mortoni*	13,991	NC_013934
	Ellobiidae	*Myosotella myosotis*	14,246	AY345053
	Pyramidellidae	*Pyramidella dolabrata*	13,856	AY345054
Patellogastropoda	Lottiidae	*Nipponacmea fuscoviridis*	18,720	MK395167
		*Lottia goshimai*	18,192	MT248298
		*Lottia digitalis*	26,835	DQ238599
	Acmaeidae	*Bathyacmaea nipponica*	16,792	MF095859
	Nacellidae	*Cellana radiata*	16,194	MH916651
		*Nacella clypeater*	16,742	KT990124
		*Nacella magellanica*	16,663	KT990125
		*Nacella concinna*	16,761	KT990126
	Patellidae	*Patella ferruginea*	14,400	MH916654
		*Patella vulgata*	14,808	MH916653
Outgroup	Mopaliidae	*Cryptochiton stelleri*	15,082	KJ569363
		*Katharina tunicata*	15,532	NC_001636

### Overlapping and noncoding regions

Most of the genes identified in *N. fuscoviridis* are located on the heavy strand except for three PCGs and seven tRNAs. In addition, fourteen genes of *L. goshimai* (seven PCGs and seven tRNA genes) are located on the light strand, with the remaining genes being located on the heavy strand (Fig. 1 and Tables 2, 3). The mitochondrial genome of *L. goshimai* contains intergenic spacers with lengths ranging from 1 to 178 bp, and there are two genes showing overlapping nucleotides (6 and 20 bp). The longest intergenic spacer is located between *trnY* and *nad5* (Table 2). The mitochondrial genome of *N. fuscoviridis* exhibits intergenic spacers with lengths ranging from 2 to 380 bp, and there are two genes with overlapping nucleotides (4 and 11 bp). The longest intergenic spacer is located between *trnY* and *nad3* (Table 3). In conclusion, there are significant differences in the intergenic spacers and overlapping nucleotides of the two species, and these species of limpets also present large variations compared with other families (e.g., Nacellidae, Acmaeidae and Patellidae)^24–28^.

**Table 2:** Annotation of the *Lottia goshimai* mitochondrial genome.

Gene	Strand	Location		Length	Codons	Intergenic nucleotide (bp)	Anticodon
		Start	Stop				
*cox1*	+	1	1560	1560	GTG/TAG	26	
*trnM1*	+	1587	1652	66		− 20	CAT
*nad1*	−	1633	2562	930	ATG/TAG	37	
*trnT*	+	2600	2668	69		3	TGT
*trnY*	−	2672	2738	67		9	GTA
*nad5*	−	2748	4421	1674	ATT/TAA	178	
*nad4l*	+	4600	4902	303	ATG/TAG	51	
*nad4*	+	4954	6279	1326	ATG/TAG	67	
*cox3*	−	6347	7204	858	ATG/TAA	10	
*trnG*	+	7215	7274	67		11	TCC
*trnS2*	+	7286	7350	65		8	TGA
*trnQ*	+	7359	7425	67		22	TTG
*atp6*	−	7448	8209	762	ATG/TAA	85	
*cox2*	−	8295	8960	666	ATG/TAA	86	
*trnL1*	+	9047	9112	66		5	TAG
*trnI*	+	9118	9189	72		8	GAT
*trnP*	+	9198	9265	68		1	TGG
*trnF*	+	9267	9334	68		43	GAA
*nad6*	+	9378	9896	519	ATG/TAA	4	
*nad3*	+	9901	10,254	354	ATG/TAA	11	
*trnM2*	−	10,266	10,332	67		45	CAT
*trnL2*	+	10,378	10,443	66		12	TAA
*trnA*	−	10,456	10,525	70		0	TGC
*trnN*	−	10,526	10,593	68		15	GTT
*trnW*	−	10,609	10,678	70		14	TCA
*nad2*	−	10,693	11,655	963	ATT/TAA	103	
*trnV*	−	11,759	11,827	69		7	TAC
*trnK*	+	11,835	11,903	69		15	TTT
*trnS1*	+	11,919	11,985	67		10	TCT
*trnD*	−	11,996	12,061	66		76	GTC
*rrnS*	+	12,138	13,058	921		11	
*trnE*	+	13,070	13,139	70		11	TTC
*trnC*	+	13,151	13,219	69		− 6	GCA
*rrnL*	+	13,214	14,746	1533		63	
*cytb*	+	14,810	15,973	1164	ATG/TAA	28	
*trnR*	+	16,002	16,070	69		1722	TCG
*atp8*	−	17,793	17,951	159	ATG/TAA	145	
*trnH*	+	18,097	18,166	70		26	GTG

**Table 3:** Annotation of the *Nipponacmea fuscoviridis* mitochondrial genome.

Gene	Strand	Location		Length	Codons	Intergenic nucleotide (bp)	Anticodon
		Start	Stop				
*cox1*	+	1	1551	1551	ATG/TAG	19	
*trnL2*	+	1571	1636	66		2	TAA
*cox3*	+	1639	2425	787	ATG/T(AA)	99	
*trnM1*	+	2525	2588	64		15	CAT
*nad4*	−	2604	3905	1302	ATG/TAA	81	
*trnC*	−	3987	4046	66		17	GCA
*nad1*	+	4064	4999	936	ATG/TAG	5	
*trnH*	+	5005	5072	68		14	GTG
*trnA*	+	5087	5153	67		84	TGC
*nad5*	−	5238	6851	1614	ATT/TAG	1562	
*atp8*	+	8413	8574	162	ATG/TAG	3	
*cox2*	+	8578	9265	688	ATG/T(AA)	115	
*atp6*	+	9381	10,181	801	ATG/TAG	41	
*cytb*	+	10,223	11,357	1135	ATG/T(AA)	68	
*trnG*	+	11,426	11,491	66		6	TCC
*trnK*	+	11,498	11,565	68		3	TTT
*trnI*	+	11,569	11,640	72		4	GAT
*trnP*	+	11,645	11,711	67		2	TGG
*trnN*	+	11,714	11,780	67		7	GTT
*trnM2*	+	11,788	11,855	68		60	CAT
*nad4l*	−	11,916	12,212	297	ATA/TAA	220	
*trnE*	+	12,433	12,499	67		58	TTC
*nad6*	+	12,558	13,046	489	ATA/TAG	4	
*trnW1*	−	13,051	13,116	66		11	CCA
*trnS1*	+	13,128	13,193	66		378	TCT
*trnL1*	−	13,572	13,637	66		2	TAG
*trnY*	−	13,640	13,706	67		380	GTA
*nad3*	+	14,087	14,440	354	GTG/TAG	− 11	
*rrnL*	+	14,430	15,867	1438		16	
*trnD*	+	15,884	15,948	65		6	GTC
*trnR*	+	15,955	16,020	66		11	TCG
*trnF*	+	16,032	16,097	66		0	GAA
*trnT*	−	16,098	16,166	69		10	TGT
*trnW2*	−	16,177	16,243	67		11	TCA
*trnQ*	+	16,255	16,321	67		− 4	TTG
*nad2*	−	16,318	17,355	1038	ATT/TAA	6	
*trnV*	−	17,362	17,426	65		129	TAC
*rrnS*	+	17,556	18,491	936		3	
*trnS2*	+	18,495	18,561	67		159	TGA

The control region (CR) is the largest non-coding region; it usually presents a high AT content and is therefore also known as the A + T rich region^29^. It is an essential element involved in mitochondrial genome replication and transcription initiation^30^. The mitogenomes of *L. goshimai* and *N. fuscoviridis* each contain one CR, and both CRs show relatively high AT contents of 61.61% and 53.43%, respectively. The CR is located between *trnR* and *atp8* in *L. goshimai,* with a length of 1722 bp. In *N. fuscoviridis,* it is located between *nad5* and *atp8,* with a length of 1561 bp. It also contains a replication origin for light-strand synthesis (OL), which is 21 bp (CCCTCCCCCCCAGGGGGAGGG) in length and folds into a hairpin secondary structure.

### Base composition of mitogenomes

The A + T content of the whole mitogenome if 60.17% for *L. goshimai* (28.18% A, 32.00% T, 24.11% G and 15.71% C), and 54.15% for *N. fuscoviridis* (23.83% A, 30.32% T, 25.39% G and 20.46% C) (Table 4). The A + T contents of all PCGs in *L. goshimai* range from 55.65% (*atp8*) to 62.64% (*cytb*), and those in *N. fuscoviridis* range from 52.07% (*nad4*) to 57.25% (*cox1*) (Table 4). We observed the highest A + T contents of the two species in PCGs (59.95% and 54.55%), followed by rRNAs (56.50% and 52.44%) and tRNAs (56.42% and 52.41) (Table 4). The AT skew of the total PCGs is negative, and the GC skew is positive across the two species, indicating that they contain a slightly higher percentage of T and G bases than A and C bases. For each PCG of two Lottiidae species in addition to the *cox2* gene of *L. goshimai*, most of the AT skew values are negative.

**Table 4:** Base composition of the mitochondrial genome of the two limpets.

Region	Size(bp)		A (%)		T (%)		G (%)		C (%)		A + T (%)		AT-skew		GC-skew	
	Lg	Nf	Lg	Nf	Lg	Nf	Lg	Nf	Lg	Nf	Lg	Nf	Lg	Nf	Lg	Nf
Mitogenome	18,192	18,720	28.18	23.83	32.00	30.32	24.11	25.39	15.71	20.46	60.17	54.15	− 0.063	− 0.120	0.211	0.108
*cox1*	1560	1551	24.49	22.63	36.15	34.62	24.04	24.37	15.32	18.38	60.64	57.25	− 0.192	− 0.209	0.222	0.140
*cox2*	666	688	31.68	25.30	27.48	27.43	16.67	28.02	24.17	19.25	59.16	52.73	0.071	− 0.090	− 0.184	0.253
*cox3*	805	787	25.59	20.08	32.55	35.58	18.88	26.94	22.98	17.41	58.14	55.65	− 0.120	− 0.272	− 0.098	0.265
*nad1*	930	936	26.13	20.73	34.52	35.04	16.99	28.10	22.37	16.13	60.65	55.77	− 0.138	− 0.257	− 0.137	0.271
*nad2*	963	1038	28.45	21.39	30.43	31.31	16.20	18.69	24.92	28.61	58.88	52.70	− 0.034	− 0.188	− 0.212	− 0.210
*nad3*	346	354	20.81	18.64	40.75	33.62	26.88	31.07	11.56	16.67	61.56	52.26	− 0.324	− 0.287	0.399	0.302
*nad4*	1326	1302	20.44	21.89	39.44	30.18	26.24	20.35	13.88	27.57	59.88	52.07	− 0.317	− 0.159	0.308	− 0.151
*nad4l*	284	297	20.77	22.90	37.32	33.33	29.58	22.90	12.32	20.88	58.10	56.23	− 0.285	− 0.185	0.412	0.046
*nad5*	1674	1614	29.57	24.10	31.66	28.62	14.22	17.97	24.55	29.31	61.23	52.73	− 0.034	− 0.086	− 0.266	− 0.240
*nad6*	519	489	18.30	23.31	43.93	32.31	24.28	30.06	13.49	14.31	62.24	55.62	− 0.412	− 0.162	0.286	0.355
*cytb*	1159	1135	21.74	20.88	40.90	34.45	20.97	25.81	16.39	18.85	62.64	55.33	− 0.306	− 0.245	0.123	0.156
*atp6*	762	801	28.35	20.72	32.28	34.58	15.49	27.59	23.88	17.10	60.63	55.31	− 0.065	− 0.251	− 0.213	0.235
*atp8*	115	162	20.00	22.22	35.65	33.33	20.87	23.46	23.48	20.99	55.65	55.56	− 0.281	− 0.200	− 0.059	0.056
tRNAs	1558	1597	28.75	24.92	27.66	27.49	24.65	26.61	18.93	20.98	56.42	52.41	0.019	− 0.049	0.131	0.118
rRNAs	2494	2374	28.47	27.38	28.03	25.06	25.54	28.52	17.96	19.04	56.50	52.44	0.008	0.044	0.174	0.199
PCGs	11,238	11,154	24.33	21.91	35.62	32.65	20.87	25.03	19.178	20.42	59.95	54.55	− 0.188	− 0.197	0.042	0.101

### Protein-coding genes and codon usage

The total length of the all PCGs is 11,238 bp in *L. goshimai* and 11,154 bp N*. fuscoviridis*, accounting for 61.77% and 59.58% of the whole genome, respectively (Table 4). The comparison of the initiation and termination codons of all PCGs showed that most of the PGCs of the two Lottiidae species are initiated with an ATN codon and terminated with TAN. Only the *cox1* gene of *L. goshimai* and *nad3* of *N. fuscoviridis* start with GTG (Tables 2, 3). While the *cox2*, *cox3* and *cytb* genes of *N. fuscoviridis* use an incomplete T stop codon, which is remarkably common in invertebrate mitogenomes.

**Figure 2:**
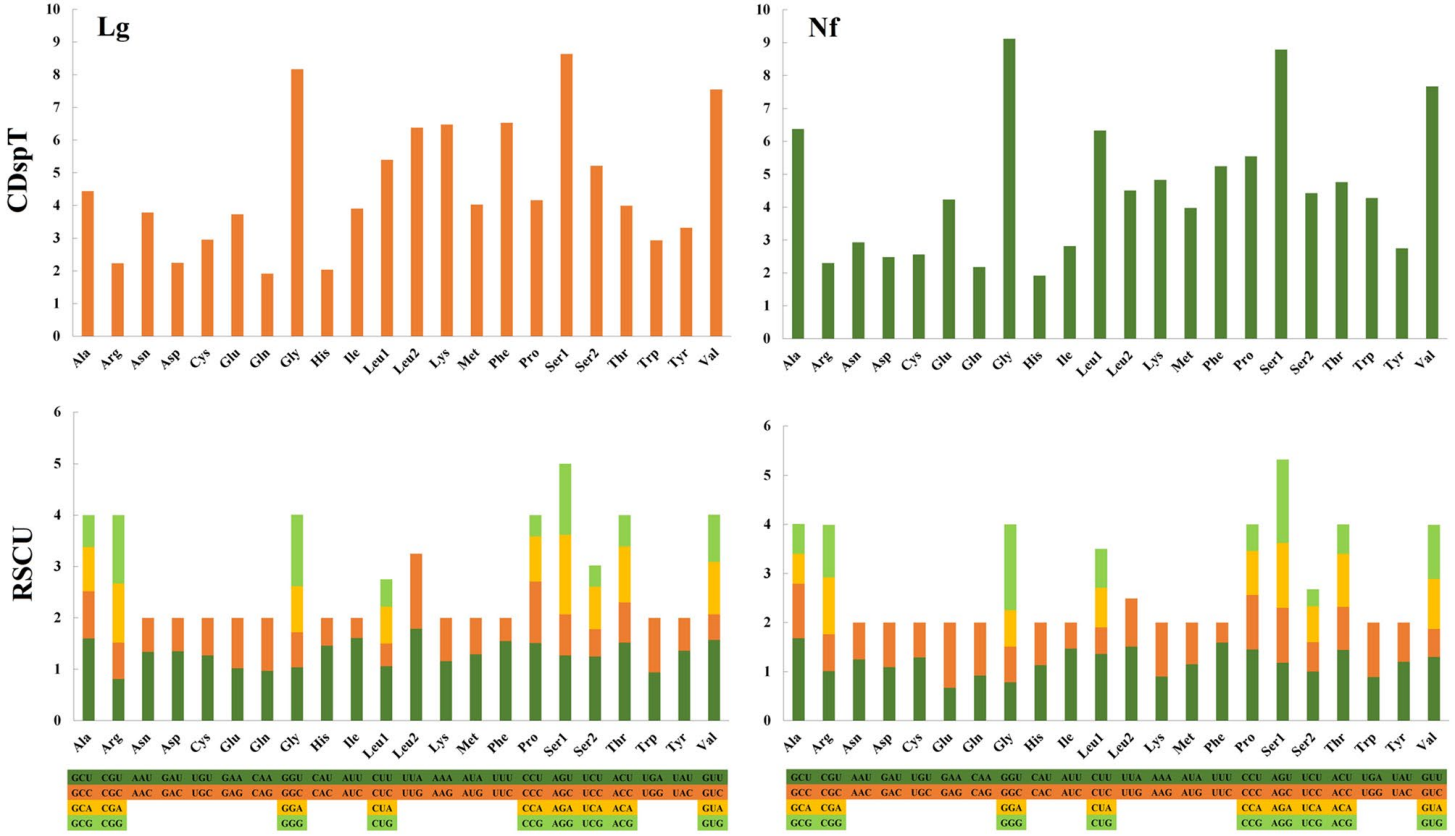
The codon distribution and relative synonymous codon usage (RSCU) in the mitogenomes of *L. goshimai* (Lg) and *N. fuscoviridis* (Nf). CDspT, codons per thousand codons.

The analysis of the two Lottiidae species indicated that the most frequently used amino acids are Gly, Ser1 and Val, while Gln and His are the least common amino acids (Fig. 2). In *L. goshimai*, the highest relative synonymous codon usage (RSCU) was found for UUA (Leu2), followed by AUU (Ile), GUU (Val) and UUU (Phe) (Fig. 2). In *N. fuscoviridis*, the highest relative synonymous codon usage (RSCU) was found for GGG (Gly), followed by GCU (Ala), UUU (Phe) and UUA (Leu2) (Fig. 2).

### Transfer RNA genes

**Figure 3:**
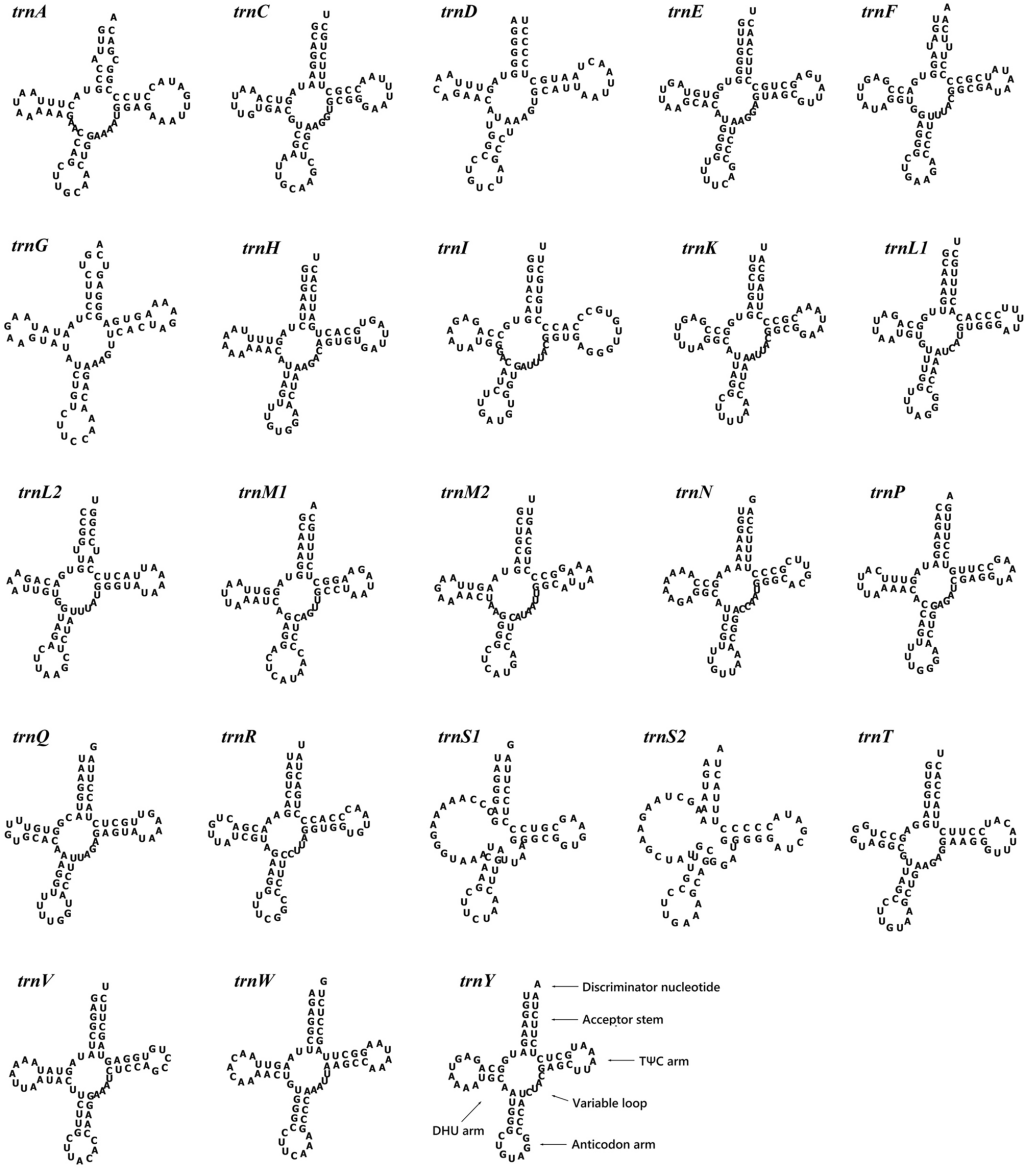
Secondary structure of the tRNA genes of the *L. goshimai* mitochondrial genome.

**Figure 4:**
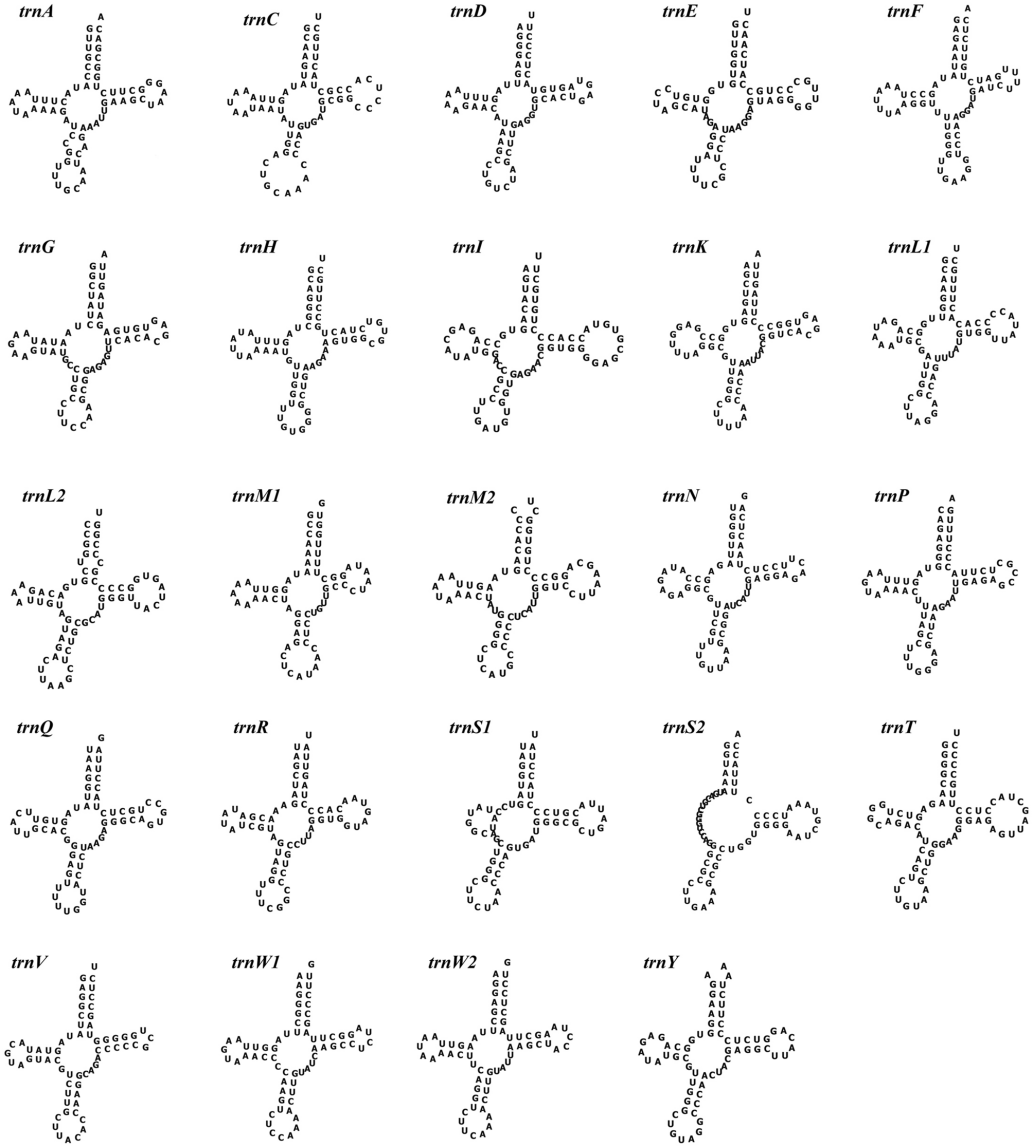
Secondary structure of the tRNA genes of the *N. fuscoviridis* mitochondrial genome.

We identified 23 tRNA genes from the mitochondrial genome of *L. goshimai*, including one more *trnM* gene than is common invertebrates, with lengths ranging from 65 (*trnS2*) to 72 bp (*trnI*). In addition, *N. fuscoviridis* exhibited one more *trnW* gene than *L. goshimai*, and 24 tRNA genes ranging from 64 (*trnM1*) to 72 bp (*trnI*) in length were identified. In both Lottiidae species, *trnS1* and *trnS2* cannot form a secondary structure due to the lack of dihydrouracil (DHU) arms, while other tRNAs are capable of folding into a typical clover-leaf secondary structure. The comparison of the tRNA genes of the two species showed that each corresponding amino acid is encoded by the same anticodon with the exception of the *trnW1* gene of *N. fuscoviridis*, which is encoded by different anticodons (CCA). Moreover, methionine is encoded by two tRNAs with the same anticodons (CAT) (Tables 2, 3 and Figs. 3, 4).

### Nonsynonymous and synonymous substitutions.

**Figure 5:**
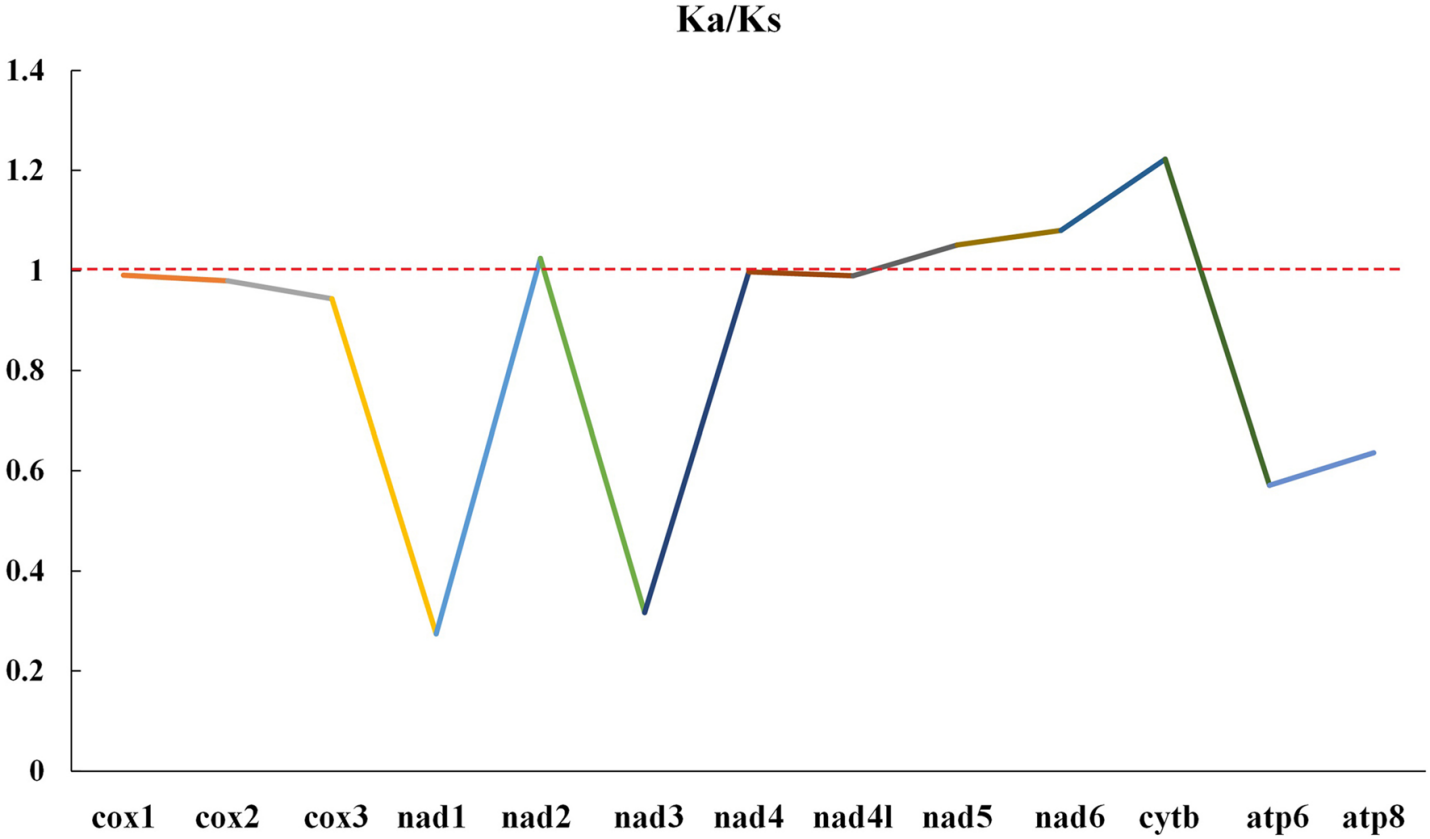
The Ka/Ks values of each PCG represent the ratios of nonsynonymous substitutions (Ka) to synonymous substitutions (Ks).

We calculated the selection pressure (estimated by using Ka/Ks) on 13 PCGs in the two Lottiidae species (Fig. 5). Most of the Ka/Ks ratios are below 1 for these PCGs, indicating that they evolved under purifying selection. The remaining *nad2*, *nad5*, *nad6* and *cytb* genes, with high Ka/Ks ratios, may have been affected by positive selection during evolution. Positive selection is influenced by the external environment for the self-regulation and transformation of genes, the elimination of genes that do not adapt to the environment, and the production of genes that can effectively adapt to the environment^31^. Therefore, advantageous genes are retained after non-synonymous mutations.

**Figure 6:**
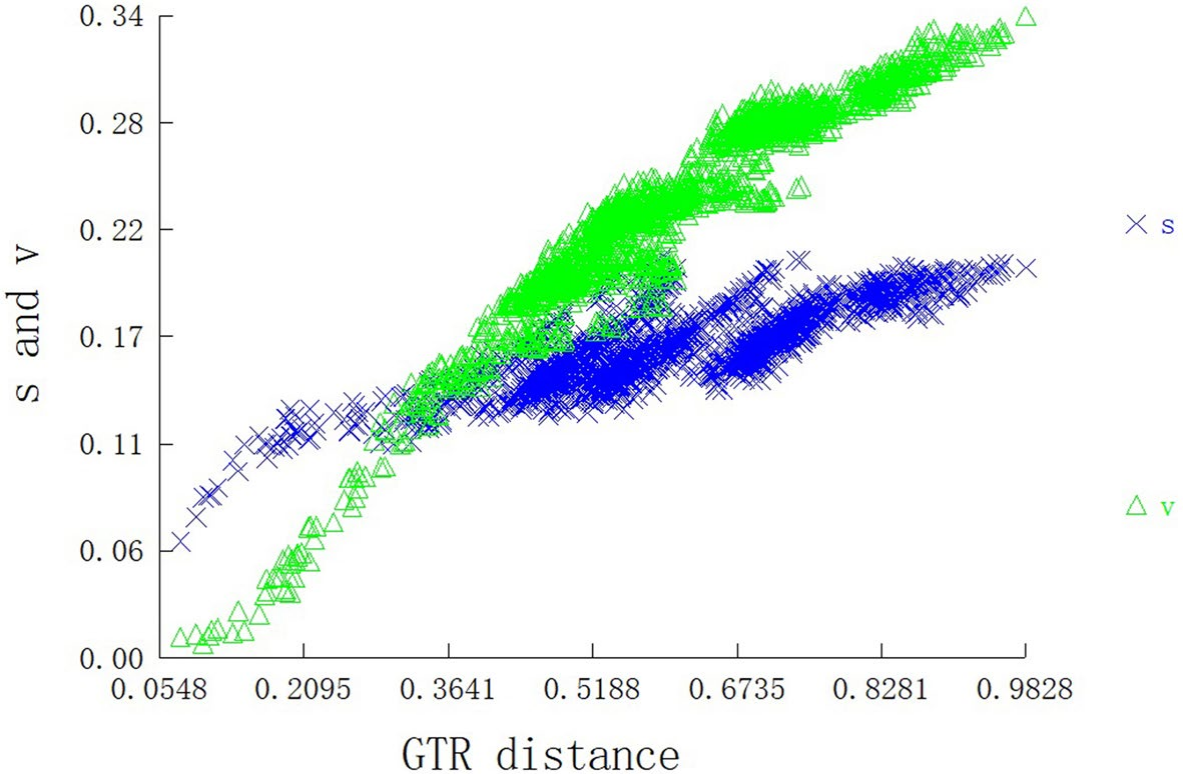
Saturation plots for all PCGs. The plots show the uncorrected pairwise divergence in transitions (s) and transversions (v) against the divergence calculated using the GTR model.

The substitution saturation index was analysed on the basis of the combined dataset of all PCGs of 60 Gastropoda mitogenomes, and the observed Iss value (Iss = 0.651) was significantly lower than that of the critical value (Iss.cSym = 0.859, *p* = 0.0000) (Fig. 6), indicating that sequence substitution is unsaturated; thus, the combined data are suitable for phylogenetic analysis.

### Phylogenetic analysis

We used the Bayesian inference (BI) and maximum likelihood (ML) methods to reconstruct a phylogenetic tree based on 13 PCGs from the two new Lottiidae species and 58 other species within Gastropoda (i.e., 8 Patellogastropoda species, 11 Caenogastropoda species, 3 Neomphalina species, 17 Vetigastropoda species, 7 Neritimorpha species, and 12 Heterobranchia species), using two Mopaliidae species as outgroups.

**Figure 7:**
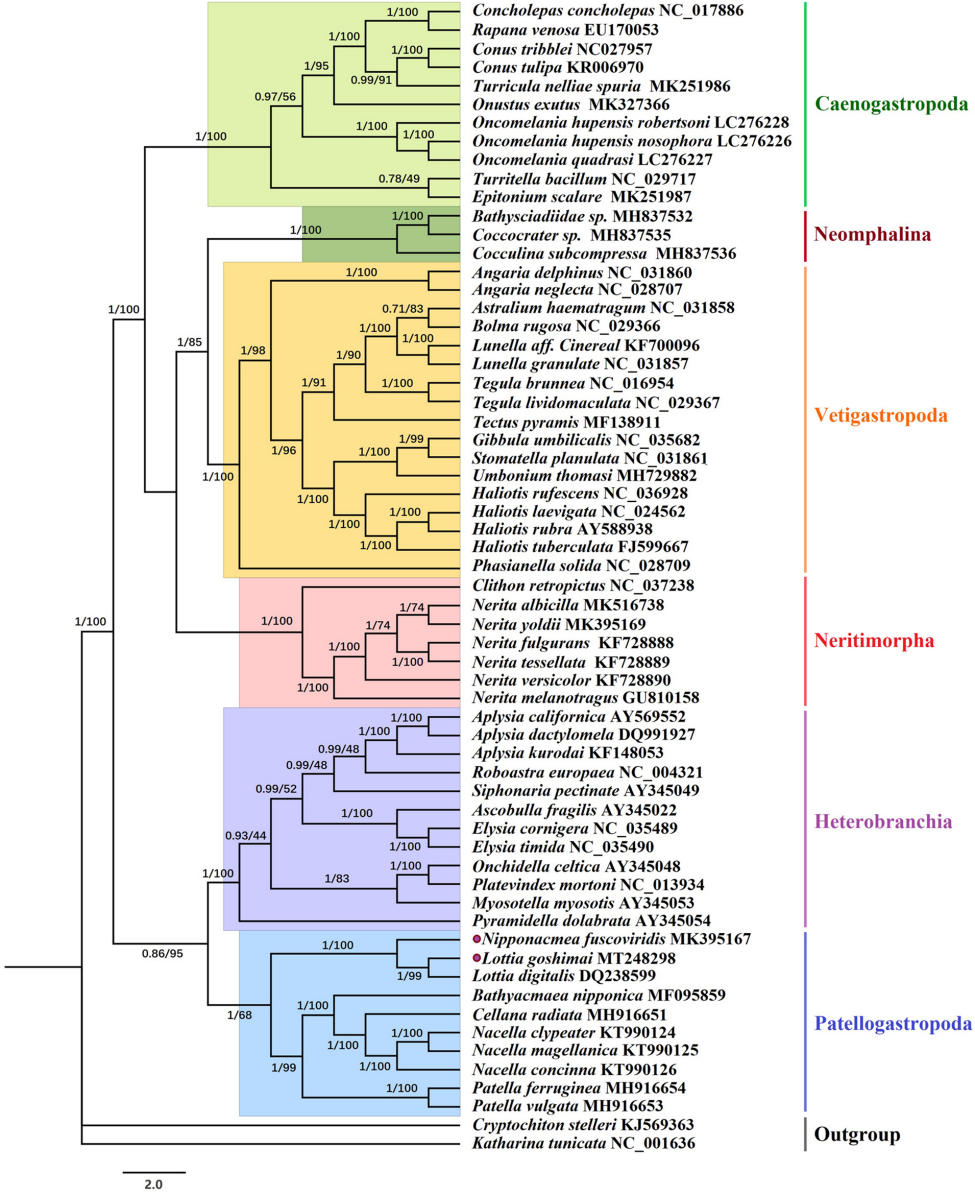
The phylogenetic tree for the two Lottiidae species and other gastropod species based on 13 PCGs. The purple dots indicate the two species sequenced in this study. The number at each node is the bootstrap probability.

In addition, in the BI analysis, due to the high rearrangement rate of Lottiidae species, which exhibited a long branch compared to other species of Patellogastropoda, we encountered a long-branch attraction (LBA) artefact in the process of constructing phylogenetic trees. This is a common systemic error in phylogenetic reconstruction resulting from the clustering of fast-evolving taxa in the tree, instead of revealing their genuine phylogenetic positions^32,33^. Specifically, the three species of the Lottiidae family and Heterobranchia erroneously formed a clade, but this situation did not appear in the ML analysis. Finally, we combined these two methods and obtained a basically consistent evolutionary tree through reference to previous research on the phylogeny of gastropods^34–37^ (Fig. 7).

The results showed a stable evolutionary tree topology in which each subclass formed a monophyletic clade. Most of the recovered clades were highly supported (Bayesian posterior probability (BPP) = 1, and Bootstrap (BS) = 100). The higher phylogenetic relationship of clade formed: (((Neomphalina + Vetigastropoda) + Neritimorpha) + Caenogastroopoda) + (Patellogastropoda + Heterobranchia). Patellogastropoda and Heterobranchia clustered together in the same clade, which was located on the outermost branch of the six subclasses. Lottiidae formed an independent branch as (*N. fuscoviridis* + (*L. goshimai* and *Lottia digitalis*)) within Patellogastropoda. *L. goshimai* was shown to be the closest extant relative of *Lottia digitalis*, and this clade clustered with *N. fuscoviridis*.

The significance of Lottiidae species in the evolution and development of gastropods was confirmed through this study. Further mitogenome sequencing work was carried out to provide more comprehensive taxon sampling for the future, thus improving the understanding of the Lottiidae phylogeny and evolution within Gastropoda.

### Gene arrangement of Patellogastropoda

**Figure 8:**
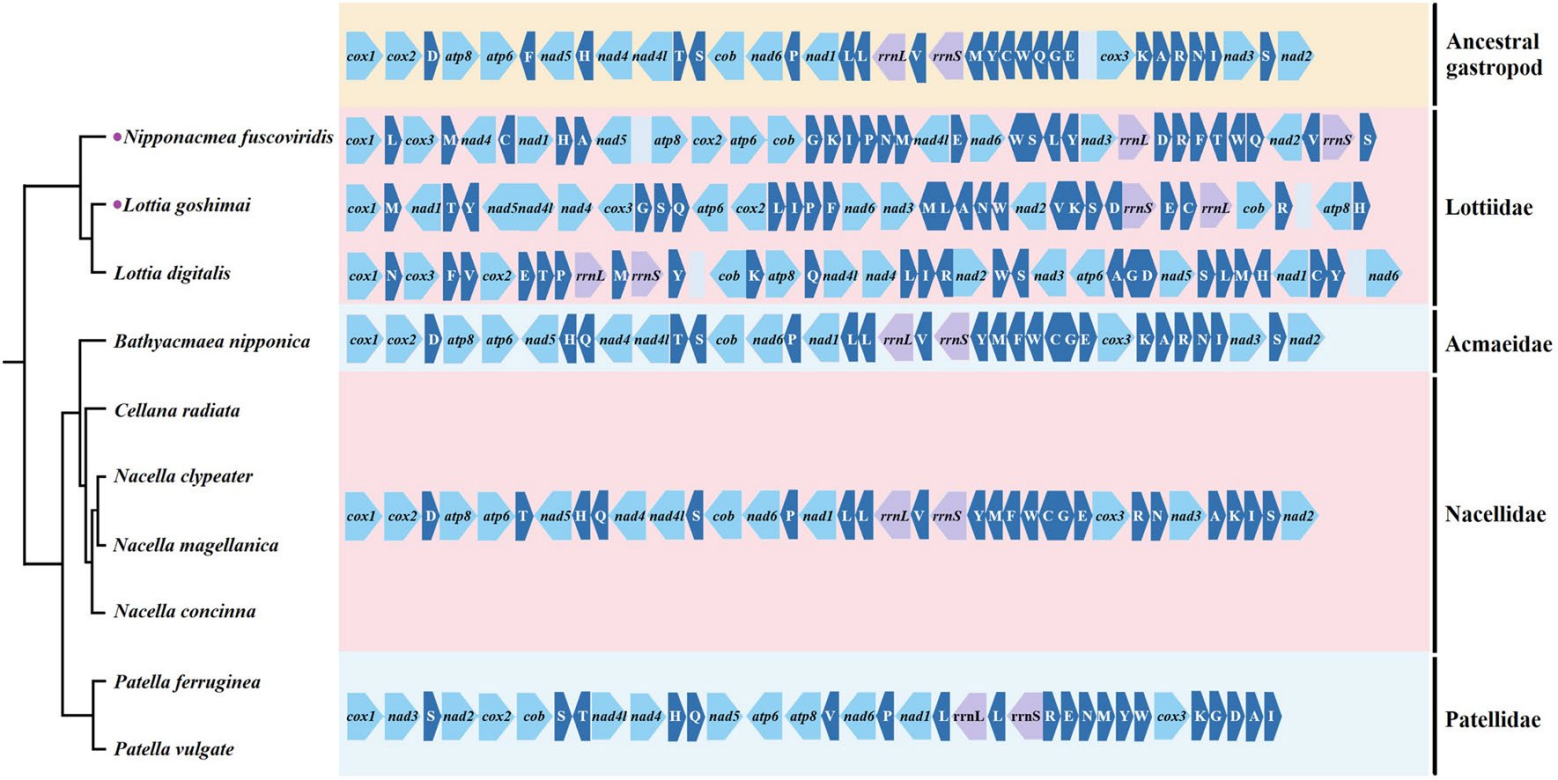
Mitochondrial genome organization of the two new Lottiidae species and available patellogastropod species.

The gene arrangements in four subclasses were compared to the hypothetical ancestral gastropod gene order^38^ (Fig. 8). Among these subclasses, the fewest gene rearrangements are observed in *Bathyacmaea nipponica* of the Acmaeidae family, and only certain tRNA sequences exhibit shuffling (*trnY* and *trnM*), translocation (*trnF*, *trnQ*, *trnF*, *trnC*) and inversion (*trnE*)^39^. The gene order is closest to that of the family Nacellidae, with six tRNAs (*trnT*, *trnR*, *trnN*, *trnA*, *trnK*, *trnI*) and one PCG (*nad3*) exhibiting translocation. Recent studies of Nacellidae mitogenomes suggest that genome rearrangements are relatively conservative in this group^11^. The phylogenetic analyses showed that Nacellidae is the sister group of Acmaeidae, which confirmed that rearrangement may be helpful for phylogenetic analysis. Compared with the above two families, the gene order in Patellidae differs substantially, but the fragment from *cytb* to *atp8* has been retained, with only a portion of this fragment exhibiting local inversion. However, the genome organization is almost the same in *Patella ferruginea* and *Patella vulgate*, indicating that they are conservative in the family Patellidae. The most noteworthy finding was that there are essential differences in gene arrangement among species of different Lottiidae families, but they share the common characteristic of *rrnL* and *rrnS* gene inversion. The mitogenomes of the Lottiidae family have retained a fraction of the clusters found in ancestral gastropods^31^. For instance, *Lottia digitalis* has retained *nad4-nad4L,* and *L. goshimai* has retained *nad5-nad4-nad4l*, with the *nad4* and *nad4l* fragments inverted in both cases. In addition, an extremely high rate of gene rearrangement is found in *N. fuscoviridis*, and the irregular ordering may be caused by a high rate of sequence evolution^40^. We will need to conduct more research on the family to verify this in the future.

## Conclusion

In this study, the complete mitochondrial genome sequences of two new limpets, *L. goshimai* and *N. fuscoviridis,* belonging to Lottiidae, were characterized and compared. Duplications of tRNA genes are found in both species (*trnM* or *trnW*). In their tRNA secondary structures, both *trnS1* and *trnS2* are missing DHU stems, which is also observed in other species of the family. The phylogenetic relationships with other members of Gastropoda based on 13 mitochondrial PCGs were analysed. The results showed that the phylogeny was consistent with morphological observations and previous reports. In addition, a highly irregular rearrangement of mitochondrial genes was found within Lottiidae. Since there are currently few species in the family, it is impossible to determine whether this situation is associated with a single species or occurs throughout the family, which is worthy of further study.

## Materials and methods

### Sample collection and DNA extraction

Specimens of *L. goshimai* and *N. fuscoviridis* were collected from the coastal area of Xiangshan County (29.48°N, 121.98°E), Zhejiang Province, in the East China Sea and Jinzhou City (40.88°N, 121.13°E), Liaoning Province, in the Bohai Sea, respectively. Morphological identification of these samples was carried out by using published taxonomic books/available taxonomic keys, and we consulted with a taxonomist from the Museum of Marine Biology of Zhejiang Ocean University^41,42^. The samples were preserved in absolute ethyl alcohol before DNA extraction. Total genomic DNA was extracted from the operculum using the salting-out method^43^ and was then stored at − 20 °C before sequencing.

### Mitochondrial genome sequencing, assembly and annotation

The whole mitogenomes of the two limpets were sequenced using the Illumina HiSeq X Ten platform (Shanghai Origingene Bio-pharm Technology Co., Ltd. China). An Illumina PE library with an insert size of 400 bp was generated. The original sequencing data have been stored in the sequence read archive (SRA, https://trace.ncbi.nlm.nih.gov/Traces/sra/) of the National Center for Biotechnology Information (NCBI). NOVOPlasty software (https://github.com/ndierckx/NOVOPlasty) was used for the de novo assembly of the clean data without sequencing adapters to obtain the optimal assembly result^44^. Two newly assembled mitochondrial genomes were annotated on the MITOS web server (https://mitos2.bioinf.uni-leipzig.de/index.py) using the invertebrate genetic code, and start and stop codons were confirmed by comparing the obtained nucleotide sequences with those from closely related limpets^24,45,46^.

### Genome visualization, secondary structure prediction and comparative studies

Circular genome visualization was conducted with the CGView Server (https://stothard.afns.ualberta.ca/cgview_server/index.html)^47^. The secondary structure of the tRNA genes was predicted using the software ARWEN (https://130.235.244.92/ARWEN/) and the tRNAscan-SE v.2.0 web server (https://lowelab.ucsc.edu/tRNAscan-SE/), as implemented on the MITOS web server^45,48,49^. The nucleotide composition and relative synonymous codon usage (RSCU) of each PCG were calculated using MEGA 7.0^50^. AT and GC skew values were calculated with the following formula: AT skew = (A − T)/(A + T) and GC skew = (G − C)/(G + C)^51^. The ratio of nonsynonymous substitutions (Ka) to synonymous (Ks) substitutions was estimated with DnaSP6.0^52^.

### Preparation of datasets, model selection, phylogenetic analyses

For the phylogenetic analysis, DAMBE 5.3.19 was used to adjust the nucleotide sequences of 13 protein-coding genes (PCGs) of each species, and the nucleotide substitution saturation was analysed to determine whether these sequences were suitable for constructing phylogenetic trees^53^. Sixty published mitochondrial genomes were downloaded from NCBI as references, including those of 58 other marine gastropods and two outgroups (*Cryptochiton stelleri* and *Katharina tunicata* of Polyplacophora), and were analysed along with the mitogenome sequence of the two new Lottiidae species (Table 1). Then, the sequences of each of 62 species were aligned using ClustalW with the default parameters in MEGA 7.0. The phylogenetic analyses incorporated Bayesian inference (BI) methods using the program MrBayes v3.2 and maximum likelihood (ML) using IQ-TREE^54,55^. MrMTgui was used to combine the results of PAUP 4.0, Modeltest 3.7 and MrModeltest 2.3 to find the best substitution models (GTR + I + G) with the AIC for Bayesian inference (BI)^56–58^. BI analyses were conducted with two Markov chain Monte Carlo (MCMC) runs, each with four chains (three heated and one cold) run for 2,000,000 generations, with tree sampling every 1000 steps and a burn-in of 25%. ML analysis was performed with the best-fit substitution model automatically selected by ModelFinder, and the number of bootstrap replicates was set to 1000 in ultrafast likelihood bootstrapping to reconstruct a consensus tree^59^. The phylogenetic trees were visualized and edited using FigTree v1.4.3^60^.

## Data Availability

The mitochondrial genome data has been submitted to NCBI GenBank under the following accession numbers: *Lottia goshimai* (MT248298), *Nipponacmea fuscoviridis* (MK395167).
